# The effect of herbal medicine in innate immunity to *Candida albicans*

**DOI:** 10.3389/fimmu.2023.1096383

**Published:** 2023-03-21

**Authors:** Meng-Yuan Bao, Ming Li, Qing-Ru Bu, Yue Yang, Hang Song, Chang-Zhong Wang, Tian-Ming Wang, Ning Li

**Affiliations:** ^1^ School of Pharmacy, Anhui University of Chinese Medicine, Hefei, China; ^2^ School of Integrated Chinese and Western Medicine, Anhui University of Chinese Medicine, Hefei, China; ^3^ Inflammation and Immune Mediated Diseases Laboratory of Anhui Province, School of Pharmacy, Anhui Medical University, Hefei, China

**Keywords:** *Candida albicans*, pattern recognition receptors, innate immune cells, herbal medicine, immunoregulation

## Abstract

*Candida albicans* (*C. albicans*) is an opportunistic pathogenic fungus that often causes mucosal and systemic infections. Several pattern recognition receptors (PRRs), such as Toll-like receptors (TLRs) and C-type lectin receptors (CLRs), have been implicated in the host recognition of *C. albicans*. These PRRs recognize the pathogen-associated molecular patterns (PAMPs) of *C. albicans* to activate innate immune cells, thereby rapidly inducing various inflammatory responses by activating intracellular signaling cascades. Herbal medicine and its active components deserve priority development due to their low toxicity and high antibacterial, antiviral and antifungal activities. This review discussed the activities of herbal compounds against *C. albicans* and their related mechanisms, especially their regulatory role on innate immune cells such as neutrophils, macrophages, and dendritic cells (DCs) implicated in *C. albicans* infections. Our work aims to find new therapeutic drugs and targets to prevent and treat diseases caused by *C. albicans* infection with the mechanisms by which this fungus interacts with the innate immune response.

## 1 Introduction

Fungal infections in hosts can range from superficial infections of the skin and mucosal surfaces to invasive infections of internal organs, resulting in a wide spectrum of diseases. Human pathogenic fungi that cause invasive infections kill over 1.5 million people each year and are therefore known as hidden killers (1). However, new antifungal drugs and diagnostic techniques for invasive fungal diseases have not been developed rapidly enough to effectively prevent or treat life-threatening fungal infections, which have resulted in significantly increased morbidity and mortality in immunocompromised patients (2). *C. albicans* is one of the common human fungal pathogens that cause invasive fungal infections. *C. albicans* is a member of the normal human microflora and is mainly found in the mucous membranes of the skin, oral cavity, gastrointestinal tract, and genital tract (3). *C. albicans* does not cause disease under normal circumstances, but it can cause opportunistic infections when the body’s immune system is altered or deficient, and when there is an imbalance in the microecology of the normal flora. *C. albicans* infections have increased significantly in recent years, especially in patients with severe immunodeficiency (4).

Innate immunity is the host’s first line of defense against fungal infection and relies on immune cells (macrophages, DCs, neutrophils, NK cells, monocytes, etc.) to defend against pathogenic fungal invasion (5). During fungal infection, innate immune cells can recognize fungal PAMPs *via* PRRs to inhibit, phagocytose and kill the fungus and initiate the innate immune response against pathogens (6). The continued use of traditional antifungal drugs (e.g., fluconazole, itraconazole, amphotericin B, etc.) and immunosuppressive drugs (e.g., the calcium phosphatase inhibitor cyclosporine A) increases the risk factors for the development of host flora disorders. Faced with a shortage of new antifungal drugs, research into the natural chemical components of herbal medicine is emerging as a source of treatment for pathogenic fungal infections, leading to a growing interest in developing new effective antifungal drugs from plants. Extract of *Curcuma longa L.* has significant antibacterial effects on bacteria and yeasts (7). In addition, the immunomodulatory proteins of *Cordyceps militaris* can promote macrophage phagocytosis *via* the TLR4-nuclear factor-kappa B (NF-κB) pathway (8).

This article reviews the PRRs and innate immune cells involved in *C. albicans* infections to comprehend how *C. albicans* interacts with innate immune cells and how *C. albicans* is restricted to the mucosal surface in normal conditions. To investigate the immune response of the host in *C. albicans* infections, as well as the prevention and treatment of *C. albicans* infections with herbal medicine, and to offer additional insights into the development of novel antifungal medications.

## 2 Recognition and response process of *C. albicans* in the host

When *C. albicans* infects the host, it first binds to PAMPs *via* PRRs on the surface of innate immune cells, triggering innate immunity, which induces the production of relevant cytokines to recruit phagocytes to kill pathogens and active adaptive immune cells to exert antibacterial effects through humoral and cellular immunity (9). TLRs and CLRs are the key PRRs used by the host to recognize *C. albicans* infections and initiate immune defense processes.

### 2.1 TLRs and CLRs

TLRs involved in the mutual recognition between *C. albicans* and the host include TLR2, TLR4, CLRs include Dectin-l, Dectin-2, Mannose Receptor (MR), and Dendritic Cell-Specific Intercellular adhesion molecule-3-Grabbing Non-integrin (DC-SIGN).

**Figure 1:**
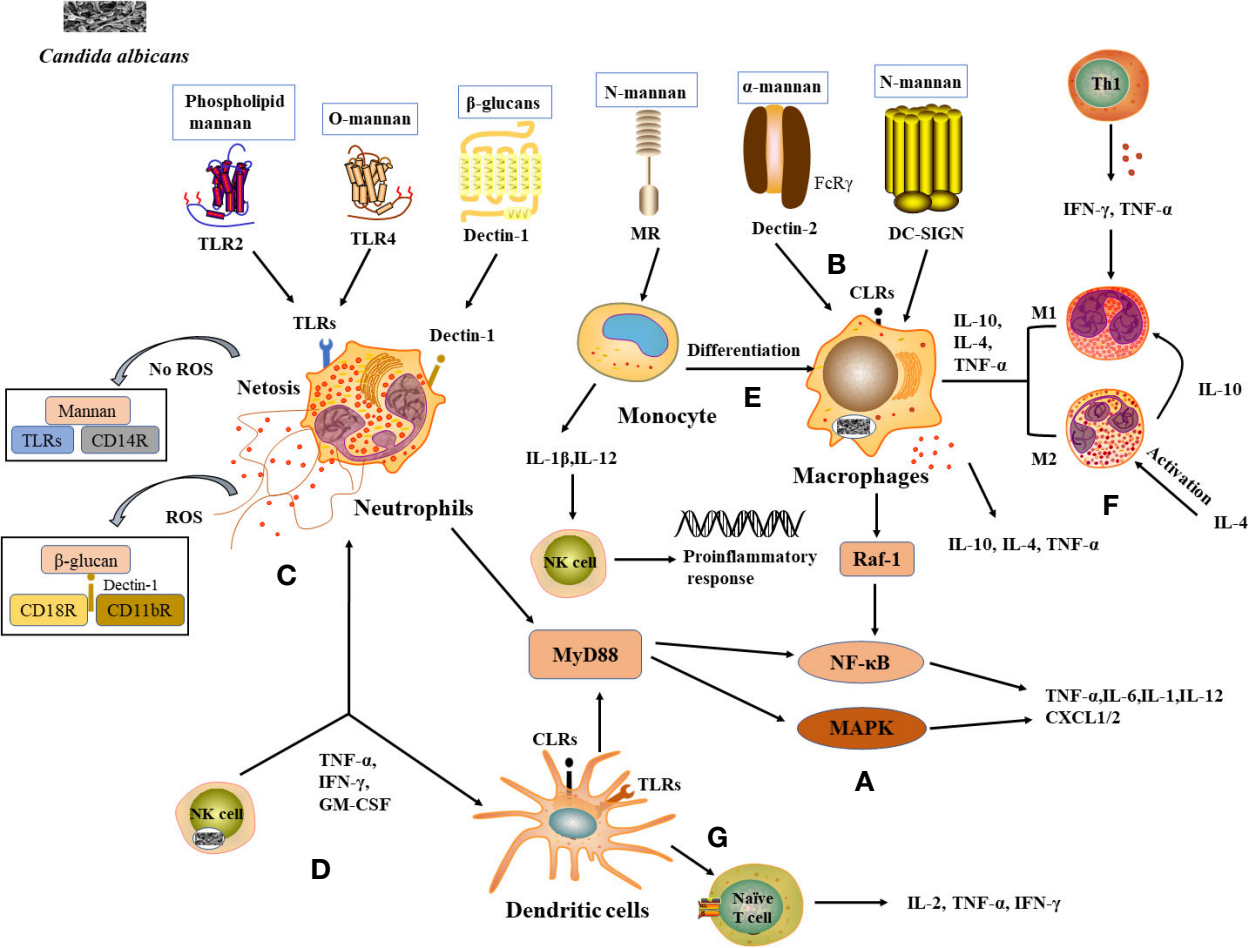
Recognition of *C albicans* cell wall components with PRRs and induction of immunocellular response. **(A)** TLRs and CLRs recognize with *C albicans* and activate immune cells to produce inflammatory factors. **(B)** PRRs on macrophages recognize **(C)** albicans cell wall components and activate corresponding signaling pathways and produces cytokines. **(C)** Neutrophils and NETs recognize signaling pathways produced by *C albicans*, thereby enhancing antimicrobial activity. **(D)** Cytokines released by NK cells enhance neutrophil activation and DCs maturation. **(E)** Monocytes differentiate into macrophages and the cytokines they produce activate NK cells to exert a stronger antimicrobial effect. **(F)** Role of differentiated macrophages M1 and M2 in the immune response. **(G)** DCs act as a bridge between innate and adaptive immunity, releasing cytokines involved in the immune response.

TLR2 and TLR4 induce an immune response by recognizing Phospholipid mannan and O-mannan components of the *C. albicans* cell wall respectively, activating the NF-κB and mitogen-activated protein kinase (MAPK) pathways, which are abundantly expressed in macrophages, neutrophils and DCs. As a result, the production of pro-inflammatory cytokines such as tumor necrosis factor-α (TNF-α), interleukins (IL-6, IL-1), and chemokines (CXCL-1, CXCL-2) that recruit and activate immune cells to clear pathogenic fungi (10) (**Figure 1A**). Dectin-1 is a type II transmembrane receptor and plays an important role in innate immunity against fungi. Dectin-1 is the most studied β-glucan receptor, mainly expressed on the surface of macrophages, monocytes, DCs, and neutrophils. It induces the release of fungal cytokines and phagocytosis (11). Dectin-2 is also a type II transmembrane receptor, mainly expressed on macrophages and DCs, and is specific for the α-mannan structure in the cell wall of *C. albicans* (12). Dectin-2 recognizes *C. albicans* hyphae and activates the NF-κB induction pathway by binding to the FcRγ receptor, resulting in phagocytosis (13) (**Figure 1B**). MR is a type I transmembrane receptor that is mainly expressed on the surface of macrophages and DCs. MR recognizes N-terminal mannose residues in the cell wall of *C. albicans* and stimulates the production of Th1-type cytokines by human monocytes to generate a Th1-type immune response (14). MR also mediates phagosome recruitment by phagocytes after the uptake of *C. albicans*, leading to the generation of cytokine *via* intracellular signaling (11). DC-SIGN increases cytokine production in DCs and macrophages by activating Raf-1 signaling *via* the NF-κB pathway (15) (**Figure 1B**).

### 2.2 Cell types involved in host immune defense against *C. albicans* infection

#### 2.2.1 Neutrophils

Neutrophils are the main immune cells that control candidiasis and are essential in the control and clearance of Candida species infections. Neutropenia is a major risk factor for invasive fungal infections, and the increased susceptibility of neutropenic individuals and mouse models to candidiasis (16). Various PRRs on the surface of neutrophils, such as TLR2, Dectin-1, etc., can recognize *C. albicans* antigenic components and thus induce neutrophil activation (17) (**Figure 1C**). Studies have shown that activation of the downstream MAPK or NF-κB signaling pathway in neutrophils leads to the expression of cytokines and antifungal factors, such as β-defensins, lysozyme, histoproteinase, which can further increase their antifungal activity (18). Neutrophils also form a neutrophil extracellular trap network (NET), which can trap and kill fungal infections (19). The formation of NET is also influenced by *C. albicans* the cell wall components mannans and β-glucan (20). Furthermore, the process of NET formation involves both reactive oxygen species (ROS)-dependent and ROS-independent signaling pathways, which can be activated by interacting with CD11b receptors (21). Among them, β-glucan triggers ROS-dependent NET production by binding to Dectin-1 as well as CD11b and CD18 receptors, whereas mannans are recognized by TLRs, CD14 receptors, and cause NET release mainly through a ROS-independent pathway (22) (**Figure 1C**).

#### 2.2.2 Natural killer cells

NK cells are essential in innate immunity against fungal invasion and can limit the further invasion and spread of *C. albicans* from the mucosal surface by promoting the immune activation of epithelial cells and phagocytes (23). NK cells can release a variety of cytokines/chemokines to modify the host immune response, such as TNF-α, GM-CSF, IFN-γ, etc., thus exerting antifungal activity in the host (24) (**Figure 1D**). It was shown that the absence of NK cells in a mouse model of systemic candidiasis leads to increased susceptibility to *Candida* spp. in mice (25). It was found that the activation of neutrophils was enhanced under the conditions of co-culture of NK cells and *C. albicans* (26). IFN-γ promotes the maturation of DCs cells and plays an important role in the Th1 cell response, a molecule that can be used as an immunotherapy for invasive fungal diseases (27) (**Figure 1D**).

#### 2.2.3 Monocyte

Monocytes circulating in the blood can infiltrate mucous membranes or inflamed tissues, where they differentiate into monocyte-derived macrophages (mo-Mac) (28) (**Figure 1E**). mo-Mac produces large amounts of pro-inflammatory molecules (such as TNF-α, IL-10, and IL-4) with potent pro-inflammatory and anti-tumor activity, which are important for inflammation, phagocytosis, and bacterial clearance (29). In the case of *C. albicans* infection, monocytes can penetrate rapidly to the site of infection and are essential in inducing a protective response (30). When mice were severely infected with *C. albicans*, monocytes and DCs were able to produce interferon (IFN). This resulted in the production of cytokine by monocytes, the activation of NK cells in response to cytokine stimulation, the secretion of inflammatory factors such as GM-CSF, the conversion of monocytes to macrophages, and the accumulation and activation of neutrophils, thus enhancing the capacity to kill *C. albicans* (31) (**Figure 1E**).

#### 2.2.4 Macrophages

Macrophages are often capable of killing *C. albicans* by phagocytosis and are considered to be key effector cells in antifungal mucosal defense. Macrophages can be divided into M1 subpopulations (classically activated macrophages) and M2 subpopulations (alternatively activated macrophages) based on their function and level of inflammatory factor secretion (32). M1-type macrophages are usually induced by Th1-type cytokines, such as IFN-γ, and TNF-α or recognized by lipopolysaccharide (LPS) and secrete high levels of IL-2 and low levels of IL-10, mainly to promote the development of inflammation, bactericidal and phagocytic effects (33, 34). M2 macrophages are mainly activated by IL-4 inflammatory factors and play a role in wound healing and tissue repair processes by inhibiting M1 macrophages through the secretion of anti-inflammatory cytokines such as IL-10 (35) (**Figure 1F**). When pathogenic microorganisms enter the body, macrophages proliferate in situ, recruiting and activating other immune cells, such as DCs and neutrophils, to the site of infection to participate in the inflammatory response (36). Macrophages recognize the cell wall components of *C. albicans* through PRRs and rely on Dectin-1-mediated phagocytosis to engulf *C. albicans* to form phagosomes, where *C. albicans* eventually lyses and die due to nutrient depletion, changes in osmotic pressure, and oxidative stress (37). Studies show that macrophage-deficient mice are significantly more likely to be infected with *C. albicans* than normal mice (38).

#### 2.2.5 Dendritic cells

DCs are the most powerful antigen-presenting cells (APCs) known *in vivo*, capable of efficient uptake, processing, and processing of antigens, initiating the body’s T-cell immune response through antigen presentation and driving the production of cytokines (IL-2, TNF-α, IFN-γ, etc.) (39) (**Figure 1G**). In addition to their powerful antigen-presenting functions, DCs also express abundant PRRs, sensitively recognize PAMPs of different types of pathogenic microorganisms, and rapidly release cytokines to participate in the immune response process, which is regarded as a bridge between the body’s innate and adaptive immunity. The Fcγ receptor family (FcγRs) plays an equally important role in the cytokine processes induced by DCs. In particular, stimulation of FcγRs alone induces little cytokine production, whereas when FcγRs act synergistically with PRRs (TLR2, TLR4), the production of pro-inflammatory cytokines increases, thereby promoting the role of DCs in the antifungal immune response (40). It was shown that mannose proteins from *C. albicans* stimulate DCs to some extent through PRRs such as TLR2 and TLR4, inducing the cytokines release from DCs and enhancing the response of helper T cells to Candida infection, including the critical Th17 response (41) (**Figure 1G**).

## 3 Innate immunoregulation and safety considerations of Chinese herbal medicine in the treatment of *C. albicans*-induced diseases

With the use of antifungal drugs, the increase in fungal resistance and the effective selection of antifungal drugs exacerbating these infections have necessitated the development of antifungal drugs. Natural products, often considered to be less toxic and biologically active, have been invaluable in the search for novel antifungal drugs (42). This special feature of the chemical composition of herbs as an important source of treatment for pathogenic fungal infection (especially *C. albicans*) may be closely linked to the bioactive substances present in them (43) (**Table 1**). As a result, there is a growing interest in developing new effective antifungal drugs derived from plants.

**Table 1:** Sources of Chinese herbal medicine and their effects on *C. albicans*-related diseases.

Product Name	Source	Diseases	Effects	References
Perillaldehyde	A natural monoterpenoid agent extracted from *Perilla frutescence*	Oropharyngeal candidiasis	Gene expression associated with adhesion of oral epithelial cells by *C. albicans* ↓ Neutropenia and reduced inflammatory response	(52, 56)
		Vulvovaginal candidiasis	Reduce neutrophils recruitment TNF-α secreted by macrophages decreases to normal levels	
Pulsatilla decoction	It is composed of 4 commonly used plants: *Radix pulsatilla* (Bai Tou Weng), *Cortex phellodendri* (Huang Bai), *Rhizoma coptidis* (Huang Lian), and *Cortex fraxini* (Qin Pi).	Vulvovaginal candidiasis	Down-regulation of Dectin-1 signaling pathway Attenuation of macrophage-stimulated ROS production Inhibition of NLRP3 inflammasome activation	(65–67, 70)
		Ulcerative colitis	Improves immune inflammatory damage of the intestinal mucosa during innate immunity	
Berberine	A quaternary ammonium alkaloid isolated from *Coptis chinensis Franch* and *Phellodendron chinense C.K.Schneid*.	Vulvovaginal candidiasis	As the main component of Pulsatilla decoction, it acts on hyphae-associated genes as well as candidalysin, further affecting the activation of neutrophils and NLRP3 inflammasome	(67, 82–86, 89)
		Ulcerative colitis	Inhibits the production of inflammatory factors in colonic macrophages and promotes apoptosis of colonic macrophages, as a candidate for exacerbation of colitis by *C. albicans*	
		Oropharyngeal candidiasis	Enters *C. albicans* cells and induces apoptosis in *C. albicans*
Paeonol	The main component of *Paeonia suffruticosa*, is derived from the Dried root bark of the peony *Paeonia Suffruticoas Andr*.	Ulcerative colitis	Down-regulation of innate immune response-related receptor-mediated signaling pathways Inhibition of NLRP3 inflammasome activation and reduction of inflammatory factors secreted by macrophages	(99, 108, 110)
		Alcoholic liver disease		
		Oropharyngeal candidiasis	Combined fluconazole/amphotericin B, reduces the hypoxic microenvironment and inflammatory response, as well as IL-17-mediated signaling
Magnolol	A lignan exacted from the bark of *Magnolia officinalis.*	Oropharyngeal candidiasis	Inhibitory effect on oral fungi, no adverse effects, and can be used as a candidate for OPC treatment	(121, 125)
		Ulcerative colitis	Improving inflammatory cell infiltration and damage in DSS-induced colonic disease, as an important reference for the treatment of exacerbated colitis by *C. albicans*	
Dioscin	A steroidal saponin extracted from the roots of *Dioscorea nipponica Makino*	Ulcerative colitis	Regulation of intestinal macrophage polarization, inhibition of colitis-related signaling pathways and activation of NLRP3 inflammasome	(138, 141)
		Alcoholic liver disease	Protects against ethanol-induced liver damage on and may be a candidate for the treatment of alcoholic liver disease aggravated by C. albicans	
Sodium houttuyfonate	Derives from *houttuynin*, which is one of the main effective phytoanticipins extracted from *Houttuynia cordata Thunb* (Saururaceae family).	Ulcerative colitis	Reduces macrophage stimulation and decreases inflammatory factor levels	(152, 155)
		Oropharyngeal candidiasis	Combined with fluconazole and reduce the expression of HIF-1α, IL-17	

### 3.1 Perillaldehyde

**Figure 2:**
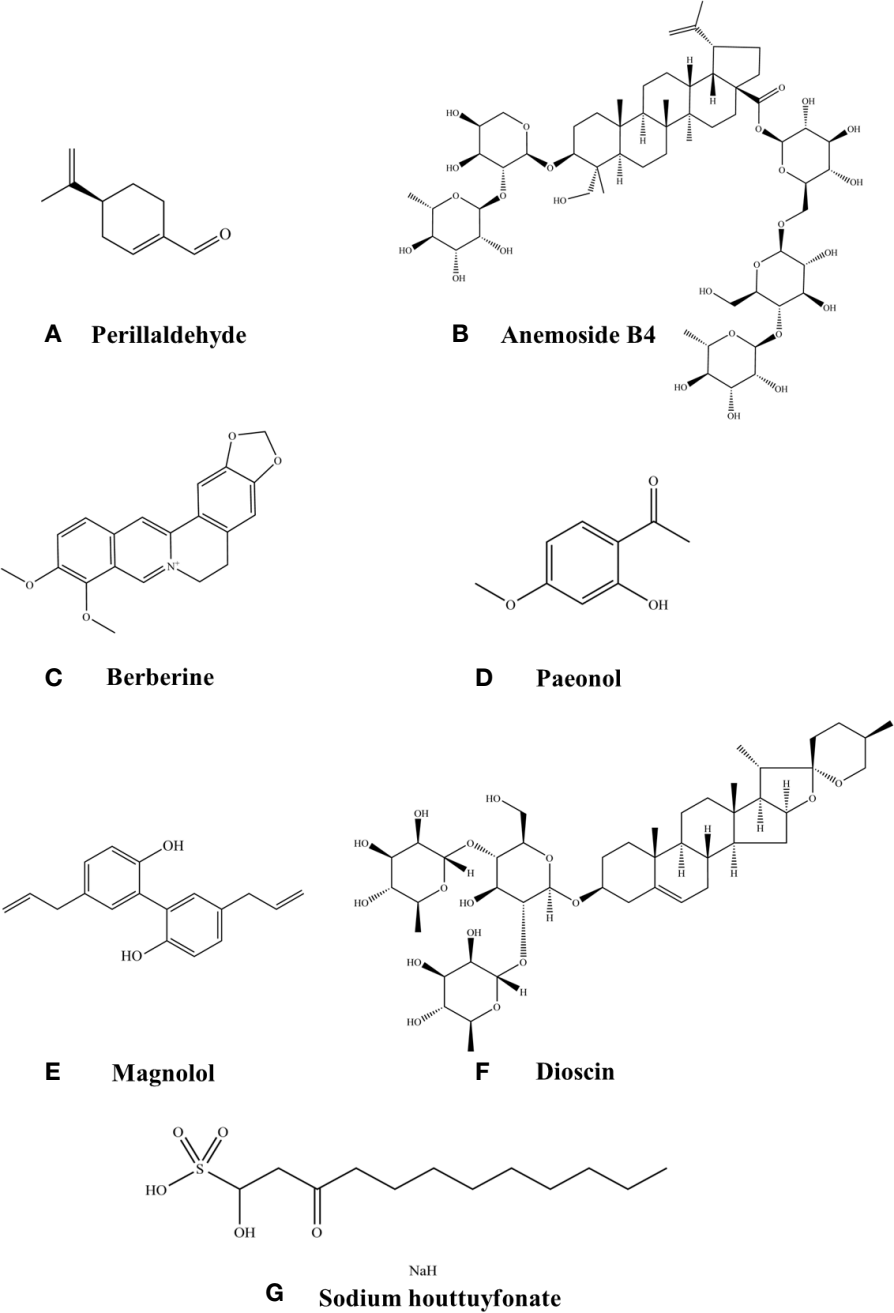
Chemical structures of the listed compounds. **(A)** Perillaldehyde. **(B)** Anemoside B4. **(C)** Berberine. **(D)** Paeonol. **(E)** Magnolol. **(F)** Dioscin. **(G)** Sodium houttuyfonate.

Perillaldehyde is a kind of therapeutic drug which is different from traditional antifungal drugs. It is a natural monoterpene compound extracted from *Perilla frutescens*. It has many biological characteristics, including anti-inflammatory, antioxidant, antiallergic, antibacterial, and reducing blood fat (44). Monoterpenes are volatile, aromatic hydrocarbons and may be considered polymers of isoprene with the formula C_5_H_8_ (The chemical structure of perillaldehyde in **Figure 2A**). Terpenes are the most abundant compounds present in herb *perilla*. Terpenes can protect plants from herbivores because of their strong odor (45).

The lipophilic nature of terpenes present in perillaldehyde allows it to be absorbed into fungal mycelium, which leads to disruption in the cell membrane of fungi (45). The mechanism of action of polyenes is to combine with cell membrane to form hydrophilic pores, which increase the permeability of the fungal cell membrane and lead to cell death. However, the disadvantage of polyenes is their high nephrotoxicity and the emergence of resistance in the organism due to long-term use (46). As a new antifungal drug, the antimicrobial activity of perillaldehyde has been gradually discovered. The study found that perillaldehyde inhibited several virulence attributes of *C. albicans* including biofilm formation, yeast-to-hyphal transition, and secreted aspartic proteinases (SAPs) gene expression (47).

#### 3.1.1 Oropharyngeal candidiasis

**Figure 3:**
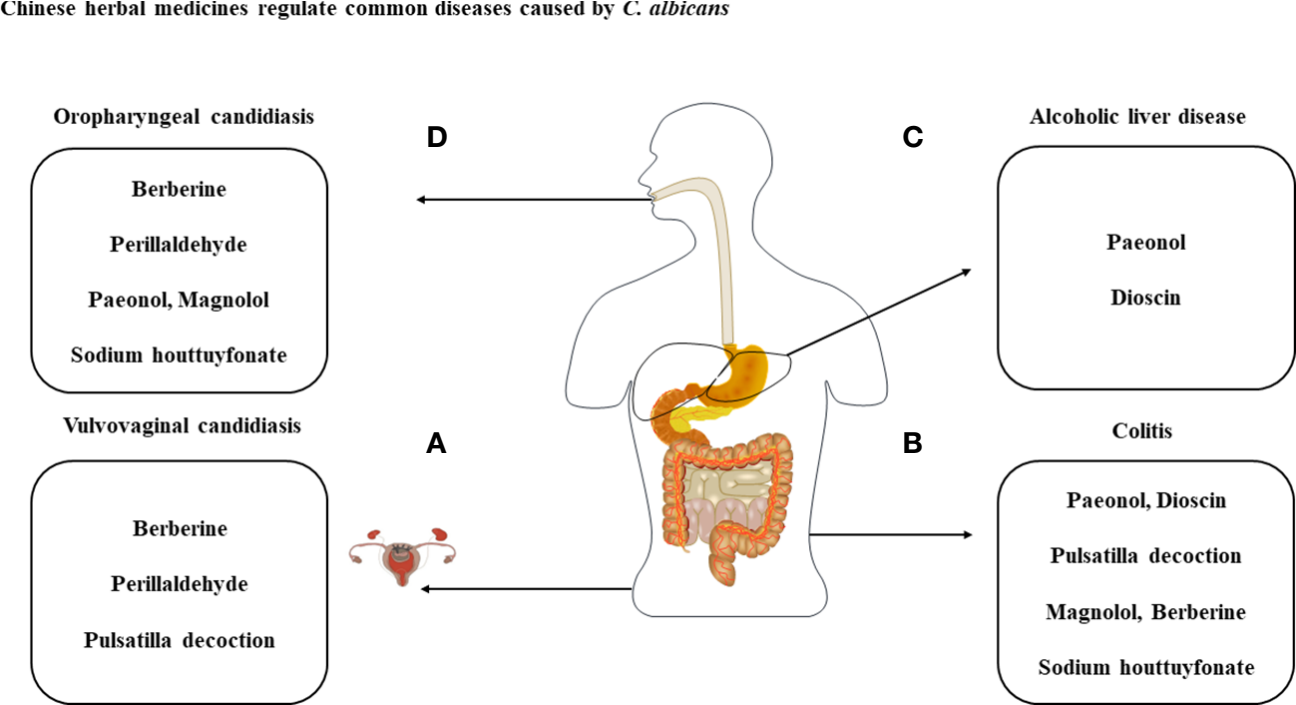
Treatment of diseases caused by *C albicans* with the listed compounds. **(A)** Perillaldehyde, pulsatilla decoction and berberine have antimicrobial effect against *C albicans* and treat VVC through cytokines produced by immune cells. **(B)** With the exception of perillaldehyde, the above-mentioned compounds have therapeutic effects on *C albicans* and DSS-induced colitis. **(C)** Dysbiosis of the intestinal flora caused by *C albicans* can exacerbate ALD and can be treated with paeonol and dioscin. **(D)** Therapeutic studies of berberine, perillaldehyde, paeonol, magnolol and sodium houttuyfonate against OPC.

Oropharyngeal candidiasis (OPC) is a common mucosal disease caused by *Candida* in oral cavity, among which infections caused by *C. albicans* are the most pathogenic (48). *C. albicans* can be parasitic in the oral cavity alone or in combination, and it does not cause disease under normal circumstances. When the oral microenvironment is stimulated by some local factors such as oral diseases, wearing dentures, or systemic factors such as using some drugs, malignant diseases, and malnutrition, *C. albicans* changes from a symbiotic organism to the pathogen, causing infection (49). Adhesion is the prerequisite for host invasion by *C. albicans* and this process is largely dependent on adhesion factors on the surface of the cell wall, of which the lectin-like sequence protein (Als) is an important adhesion-related gene in *C. albicans* and plays a key role in adhesion and biofilm formation. The study found that the aggressiveness and virulence of Als3 deletion mutant strain on host mucosal epithelial cells were significantly reduced (50). The mycelial cell wall protein Hwp1 is a hyphae-associated GPI-dependent protein that binds to dextran on the surface of the cell wall through covalent bonding, and Hwp1p mediates the adhesion of *C. albicans* to oral mucosal epithelial cells (51). It was found that the mRNA levels of Als3 and Hwp1 gradually decreased with increasing concentrations of perillaldehyde (47). Various PRRs on the surface of neutrophils induce neutrophil activation by recognizing the antigenic component of *C. albicans* (17). The experimental results showed that a large number of mycelium colonized the surface of the tongue tissue and that the host recruited a large number of neutrophils to remove microorganisms. With increasing concentrations of perillaldehyde, neutrophils on the tongue tissue gradually decreased and had significant bactericidal and anti-inflammatory effects in OPC (47) (**Figure 3D**).

#### 3.1.2 Vulvovaginal candidiasis

Perillaldehyde is also therapeutic vulvovaginal candidiasis (VVC) caused by *C. albicans* (**Figure 3A**). VVC is a common gynecological fungal infectious disease. VVC is mainly caused by *C. albicans* infection of the vaginal mucosa (52). More than 75% of women have VVC at least once in their lives, and 5% to 10% of them have VVC more than 4 times a year, that is, recurrent vulvovaginal candidiasis (RVVC) (53). *C. albicans* colonizes different parts of the host and resulting in tissue damage. Neutrophils are attracted to sites of cell damage in different tissues, where they produce dense populations. The powerful weapon of neutrophils is their ability to resist pathogens and cause massive collateral damage, further prolonging the activation of immune cells, the loss of functional tissue and ultimately organ dysfunction (54). Studies have found that the colonization of *C. albicans* recruited a large amount of neutrophils into the vagina, but after treatment with perillaldehyde, the neutrophils in tissues decreased significantly, accompanied by the gradual recovery of tissue integrity (44). Similarly, *C. albicans* introduces a large number of macrophages to aggregate in the vagina, and TNF-α is mainly secreted by macrophages (55). Experimental studies have shown that TNF-α concentration were significantly increased after *C. albicans* infection, and perillaldehyde treatment reduced TNF-α concentration to normal levels (44). Furthermore, perillaldehyde was found to have anti-inflammatory activity in a mouse model of dextran sodium sulfate (DSS)-induced colitis. The perillaldehyde administration resulted in suppression of DSS-induced expression of pro-inflammatory cytokine genes and matrix metalloproteinase-9 in the colon, these effects that have been demonstrated in lipopolysaccharide-stimulated macrophage RAW264.7 cells. And amelioration of intestinal inflammation *in vivo via* JNK-mediated cytokine regulation (56). Perillaldehyde can also ameliorate *A. fumigatus* keratitis by activating the Nrf2/HO-1 signaling pathway and inhibiting the Dectin-1 mediated inflammatory response and neutrophils recruitment (57). Perillaldehyde provides a new strategy and theoretical basis for the clinical study and treatment of inflammatory diseases and antifungal drugs.

#### 3.1.3 Toxicity of perillaldehyde

The toxicity of perillaldehyde is related to the dose used. Studies have found that perillaldehyde treatment at a moderate dosage (10 mg/kg-100 mg/kg) is likely not to cause any damage or weight loss to delicate organs of the body like the liver, kidney, and spleen, side effects and suppressed efficient penetration may occur at higher concentrations (56). In lieu of these findings and concerns on genotoxicity in the liver, EFSA made a logical conclusion that perillaldehyde demonstrated genotoxic potential *in vivo* and therefore posed a potential safety concern as a flavoring substance (58).

### 3.2 Pulsatilla decoction

Pulsatilla decoction, was first prescribed by Zhang Zhongjing in “Shang Han Lun”, approximately 1800 years ago. It is composed of 4 commonly used plants: *Pulsatilla chinensis* (Bai Tou Weng), *Cortex phellodendri* (Huang Bai), *Rhizoma coptidis* (Huang Lian), and *Cortex fraxini* (Qin Pi). *Pulsatilla chinensis* is the chief herbal source of Pulsatilla decoction, and it is rich in triterpenoid saponins, such as anemoside B4 (AB4), anemoside A3 (AA3), and 23-hydroxybetulinic acid. AB4 is the most abundant of that group and has been used as a quality control marker for Pulsatilla chinensis (59) (The chemical structure of anemoside B4 in **Figure 2B**).

#### 3.2.1 Vulvovaginal candidiasis

*Pulsatilla chinensis*, another traditional Chinese medicine rich in Pentacyclic triterpenoid saponin (PTSs), and saponins may be the main components of its antibacterial activity (60). Sugars are attached to one or more points of this structure, forming chains that could be branched. This appearance leads to amphiphilic properties giving saponins the ability to interact with both lipophilic and hydrophilic structures (61). The surfactant behavior lets them interact with biologic membrane layer, this action may perturb the membrane and its function leading to membrane perforation or complete lysis. The formation of *C. albicans* biofilms is one of the virulence factors, indicating that pulsatilla decoction has great potential in inhibiting biofilm and thus exerting therapeutic effects. Dectin-1 as the primary PRR for recognizing β-glucan, dectin-1^-/-^ mice are susceptible to *C. albicans* infection, which is associated with impaired cytokine production and poor neutrophil-mediated fungal eradication (62). It was demonstrated that the Dectin-1-Syk-CARD9-NF-κB signaling pathway was activated in VVC mouse models and after pulsatilla decoction administration, it effectively reduced fungal infection and down-regulated the Dectin-1 signaling pathway, which has application value in improving the clinical treatment of VVC (63). Dectin-1 expressed on the surface of macrophages recognizes *C. albicans* cell wall component β-glucan and then initiates the appropriate signaling pathways to generate an inflammatory response to clearance pathogens, such as activation of nucleotide oligomerization domain-like receptor family pyrin domain ontaining 3(NLRP3) inflammasome (64). Macrophage stimulation by *C. albicans* also leads to the production of ROS, which act as intermediate triggers to activate NLRP3 inflammasome, exacerbating the subsequent inflammatory cascade response and cellular damage. Our research group showed that serum containing n-butanol extract of Pulsatilla decoction can reduce the expression of NLRP3 protein and inflammatory cytokines, and inhibit the release of ROS to inhibit the activation of NLRP3 inflammasome (65), thus exerting therapeutic efficacy in the treatment of VVC (**Figure 3A**). It provides a solid foundation for the further development of novel antifungal drugs for VVC.

#### 3.2.2 Ulcerative colitis

Inflammatory bowel disease (IBD) is composed of Crohn’s disease (CD) and ulcerative colitis (UC). UC is a chronic non-specific inflammatory disease that mainly accumulates in the rectum and colonic mucosa. The occurrence and development of UC are related to a variety of potential pathogenic factors, such as intestinal microbial ecological disorders and intestinal immune dysfunction (66). It is reported that the metabolic pathways and molecular mechanisms involved in *C. albicans*-related ecological disorders play a key role in the intestinal mucosal barrier function and innate immunity of UC (67). In patients with UC, *C. albicans* overproliferates and its antigens or metabolites abnormally activate immune cells to release cytokines to cause immune response or inflammation, resulting in worsening of the intestinal condition of the patients. Experimental studies have shown that n-butanol extract of Pulsatilla decoction was effective in reducing the expression of cytokines IL-1β, IL-6, IL-8, and defensins HBD-2 and HBD-3 after treatment of UC mice under over-colonization by *C. albicans* (68) (**Figure 3B**). The results shown that n-butanol extract of Pulsatilla decoction not only directly inhibited *C. albicans* in the intestinal tract, but also improved the immune inflammatory damage of the intestinal mucosa involved in the innate immune process caused by the colonization of *C. albicans*, which would provide a good foundation for the treatment of *C. albicans*-associated intestinal diseases by n-butanol extract of Pulsatilla decoction. In addition, pharmacological studies have shown that as the main chemical constituent of pulsatilla decoction, anemoside B4 has significant anti-inflammatory and immunomodulatory activities *in vivo* (59). Studies have shown that Anemoside B4 can ameliorate LPS-induced kidney and lung inflammation damage, which inhibited pro-inflammatory response by NF-κB pathway in mice (69). Cisplatin (cis-Dichlorodiammine platinum, CP), as the first-line chemotherapy drug of choice for many cancers, also causes toxicity and side effects to the kidney. In addition, anemoside B4 showed significant protective effect on acute kidney injury induced by cisplatin in mice. It mainly acted on NF-κB signaling pathway to reduce the levels of TNF-α, IL-1β, cyclooxygenase-2 (COX-2) and inducible nitric oxide synthase (iNOS), thus exerting anti-inflammatory activity (70). Anemoside B4 also ameliorates 2, 4, 6-trinitrobenzene sulfonic acid (TNBS)-induced colitis symptoms. Quantitative proteomic analyses discovered that 56 proteins were significantly altered by anemoside B4 in TNBS-induced colitis rat model, among all the proteins, S100A9 is one of the most significantly down-regulated proteins and associated with NF-κB and MAPK signaling pathways in the pathogenesis of ulcerative colitis. In addition, anemoside B4 suppressed the expression of S100A9 downstream genes including TLR4 and NF-κB in colon tissue (71). This provides an important theoretical basis for pulsatilla decoction and its main ingredient anemoside B4 in treating colitis-related diseases.

#### 3.2.3 Toxicity of pulsatilla decoction

Pulsatilla decoction showed considerable cytotoxic activity to human cancer cell lines (A549, SGC-7901) and human hepatic cell line (HL-7702) (150). The effect of anemoside B4 effect was accompanied by a downregulation of the drug efflux pump multidrug resistance protein 1 (MDR1), increasing intracellular drug accumulation. In the *in vitro* experiment anemoside B4 itself shows an inhibitory effect on MDR1; however, after *in vivo* biotransformation its metabolites are present, and they may have an opposing effect, which is dominant in the *in vivo* system. These observations raise the possibility that coadministered anemoside B4 may reduce the therapeutic effects of other drugs, which could result in therapeutic failure (151). Therefore, anemoside B4 should be used with caution to prevent its harmful interactions and enhance its beneficial drug interactions.

### 3.3 Berberine

Berberine (BBR), a quaternary ammonium compound (The chemical structure of berberine in **Figure 2C**), is the most abundant bioactive component found in traditional Chinese herbs *Coptis chinensis Franch* [Ranunculaceae] and *Phellodendron chinense C.K.Schneid*. [Rutaceae]. Berberine can play the role of antibacterial, antifungal, anti-inflammatory, immune modulator, etc (86).

As far as antifungal is concerned, berberine can either directly inhibit *C. albicans* or act as a sensitizer to enhance the antibacterial effect of FLU against *C. albicans*. *C. albicans* is susceptible to resistance to commonly used antifungal drugs such as fluconazole and amphotericin B (72). Berberine was found to inhibit fluconazole-resistant *C. albicans* through its action on the high-osmolarity glycerol mitogen-activated protein kinase (HOG-MAPK) pathway, with increased susceptibility to fluconazole after treatment (73). The formation of *C. albicans* biofilm increases its drug resistance and virulence. After berberine intervention, the entire structure of the biofilm was disrupted and the expression of key regulatory genes during biofilm formation was significantly reduced, thereby inhibiting mycelial cell formation and proliferation (74).

#### 3.3.1 Vulvovaginal candidiasis

The hyphae-phase *C. albicans* exhibits greater invasiveness than the yeast phase due to the release of the novel toxin candidalysin and hydrolytic enzymes such as SAP, which are closely related to its cell wall (75, 76). It was found that as the concentration of berberine increased, the mycelial phase gradually decreased and some damaging effects on the cell wall of *C. albicans* could be clearly observed (77). The *C. albicans* mycelium-specific gene ECE1 is not only expressed in the mycelial phase of *C. albicans* to be responsible for mycelial elongation, but also encodes for the production of candidalysin in the mycelial state (75). Candidalysin plays an important role in VVC through recruiting neutrophils and activating NLRP3 inflammasome (78, 79). In our research, n-butanol extract of Pulsatilla decoction has therapeutic effects on VVC (65). Berberine, as the main component of n-butanol extract of Pulsatilla decoction, suggests the possibility of reducing the activation of neutrophils recruitment to NLRP3 inflammasome by inhibiting ECE1 gene expression, which has important reference value for the treatment of VVC (**Figure 3A**). Furthermore, our research group found that berberine plays a key role in preventing *C. albicans* from adhering to vaginal epithelial cells (80). After berberine intervention, the expression of ICAM-1 and mucin related to *C. albicans* adhesion was significantly reduced, which had a certain therapeutic effect on VVC (80).

#### 3.3.2 Ulcerative colitis

Berberine is also an effective drug for the treatment of DSS-induced colitis. Berberine inhibited proinflammatory cytokine production, including TNF-α, IL-17 and IFN-γ, in colonic macrophages and epithelial cells in DSS-treated mice and promoted apoptosis of colonic macrophages. Berberine administration dramatically decreased ILC1 and Th17 cells, and increased Tregs as well as ILC3 in colonic tissue of DSS-induced mice, and it was able to regulate the expression of various immune factors at the mRNA level (81). *C. albicans* colonization exacerbates the deterioration of intestinal conditions in UC patients. Berberine may be a potential candidate for the treatment of colitis exacerbated by *C. albicans* colonization (**Figure 3B**). Furthermore, berberine inhibited proliferation of colon cancer cells in a dose- and time- dependent manner. Chidambara et al. (82) have demonstrated that berberine can induce apoptosis in colon-cancer cells, through the induction of a series of biochemical events, including loss of mitochondrial-membrane potential, release of cytochrome-c to cytosol, induction of Bcl-2 family proteins and caspases, and cleavage of poly ADP ribose polymerase (PARP). Berberine also can induce autophagic death in colon-cancer cells and hepatic carcinoma cells by elevating glucose regulated protein 78 (GRP78) (83). These studies suggest that berberine is important in the treatment of colon-related diseases.

#### 3.3.3 Oropharyngeal candidiasis

Studies have shown that berberine can enter *C. albicans* cells and function both extracellular and intracellular. Berberine treatment decreases ergosterol, resulting in loss of membrane permeability and inducing cell death in *C. albicans* cells. The accumulation of berberine at a dose of 50 μg/ml is time-dependent and suggests that berberine may be used as an alternative therapy for the treatment of candidiasis (84, 85) (**Figure 3D**), which also provides important reference value for the treatment of OPC with berberine.

#### 3.3.4 Toxicity of berberine

The administration method is a significant factor which can affect acute toxicity of berberine. Berberine has poor absorption in the gut, and most of the oral dose remained inside the gastro-intestinal lumen, which was excreted in the feces finally. The part of berberine which was absorbed into body could be converted into multiple metabolites. In fact, berberine and its metabolites exist simultaneously *in vivo* (86). Some other clinical studies indicated different adverse effects such as transient elevation in serum bilirubin level (87). Therefore, the clinical application of berberine still requires careful consideration.

### 3.4 Paeonol

Paeonol (PAE, 2-hydroxy-4-methoxy acetophenone) (The chemical structure of paeonol in **Figure 2D**) is a natural phenolic extract from Paeonia *suffruticosa* bark and is one of the main active ingredients in Chinese herbal medicines such as *Pulsatilla*, *Paeoniae Radix Alba* and *Paeonia. lactiflora Pall*. Paeonol has a variety of pharmacological effects such as anti-inflammatory, blood lipid lowering, anti-oxidation and anti-tumor (88).

Phenolic compounds also possess a strong ability to bind to different molecular structures, such as proteins or glycoproteins and may produce antimicrobial effects by inhibiting DNA/RNA transfer and protein transport (89). The disruption of cell walls and membranes by plant polyphenols causes the leakage of alkaline phosphatase, ATP, proteins and other nutrients, resulting in irreversible damage to cell morphology (90). Paeonol could be considered a promising source of novel natural antimicrobial agents and anti-biofilms for use in a variety of pharmaceutical products. Our group demonstrated that paeonol had moderate antifungal potential and strong synergism with fluconazole/amphotericin B by inhibiting fungal growth and virulence factors in *C. albicans*, increased survival of *C. albicans*-infected hosts and reduced fungal burden (91).

#### 3.4.1 Ulcerative colitis

The study found that the application of paeonol enema attenuated trinitrobenzene sulfonic acid-induced colitis through inhibiting mRNA expression induced by costimulation of TNF-α and IFN-γ *via* MAPKs/NF-κB/STAT1 pathway (92). Paeonol and anemoside B4 achieve the same goal with different means. Clinical practice shows that Paeonol is effective in the treatment of ulcerative colitis (UC) (93, 94), but it has not been shown to be effective in the treatment of colitis with *C. albicans* pre-colonization. Our group previously studied to establish a DSS-induced colitis model with *C. albicans* pre-colonization and found that the introduction of *C. albicans* further exacerbated DSS-induced colitis, with local and systemic inflammation significantly higher than in the DSS group (95). The host’s innate immune response to *C. albicans* is controlled by several receptor-mediated signaling pathways, including Dectin-1, TLR2 and TLR4, and synergistic effects between them are essential. Under the intervention of paeonol, it was found that the fungal load in feces and organs decreased, and the expression of Dectin-1, TLR4, TLR2, and NF-κB decreased significantly (95) (**Figure 3B**). Excessive pro-inflammatory factors and insufficient anti-inflammatory factors play a key role in the progression of IBD (96). In addition to its potential role in regulating the intestinal microbiota, paeonol inhibits the endotoxin-induced pro-inflammatory cytokines TNF-α, IL-1β and IL-6 and elevates the anti-inflammatory cytokine IL-10 in macrophages (97). Therefore, pae could be a candidate for the treatment of ulcerative colitis associated with fungal ecological disorders.

#### 3.4.2 Alcoholic liver disease

Epirubicin is widely used for the treatment of various breast cancers; however, it has serious adverse side effects, such as hepatotoxicity, which require dose-adjustment or therapy substitution. Paeonol attenuates epiamine-induced hepatotoxicity by inhibiting the PI3K/Akt/NF-kB pathway, thereby exerting some hepatoprotective effects (98). Alcoholic liver disease (ALD) is a common clinical liver disease, which is mainly caused by inflammatory damage caused by a large amount of alcohol for a long time, which is characterized by an imbalance of intestinal flora and excessive colonization after fungal infection, thus increasing the permeability of intestinal mucosa (99). The predominant commensal fungal species in the human intestine are Candida species, Saccharomyces cerevisiae, and Malassezia species (100). Like commensal bacteria in the intestine, fungi interact with their host. Although the host’s immune system develops tolerance to colonization with commensal fungi, it must contain the spread of the fungi, especially invasion (101). The intestinal barrier is the first line of defense against fungal PAMPs. More and more evidence show that intestinal barrier dysfunction is involved in the pathogenesis of ALD by promoting liver inflammation (102). Studies have shown that overgrowth of *C. albicans* and mycotoxin *C. albicans* (candidalysin) produced by its hyphae can aggravate ALD by activating NLRP3 inflammasome (99). Our research group has demonstrated through *in vitro* and *in vivo* experiments that impaired intestinal barrier function and weakened immunity lead to overgrowth of C. albicans, resulting in the release of β-glucan into the bloodstream, entering the liver through the portal vein and activating the Dectin-1/IL-1β signaling pathway. After paeonol intervention, it can inhibit β-glucan, block the binding of Dectin-1 to it, and then inhibit the activation of the NLRP3 inflammasome, thus reducing the levels of IL-1β and IL-18 secreted by macrophages, alleviating ALD inflammatory injury (103) (**Figure 3C**). These studies suggest that paeonol has important reference value in the treatment of liver disease.

#### 3.4.3 Oropharyngeal candidiasis

It is reported that paeonol has preventive potential in the occurrence of oral cancer (104). Based on the research of our group, paeonol has been shown to alleviate inflammation in DSS-induced colitis under *C. albicans* pre-colonization and alleviate the ALD of mice aggravated by *C. albicans via* the Dectin-1/IL-1β signaling pathway. On this basis, it was investigated whether paeonol has a therapeutic effect on OPC. Many clinical strains of *C. albicans* are resistant to fluconazole/amphotericin B and exhibit multi-drug resistance. We observed that paeonol combined with fluconazole/amphotericin B was effective in reducing the colonization of *C. albicans* in OPC mice and improving the integrity of the mucosal surface (105) (**Figure 3D**). When *C. albicans* is present on the surface of the oral mucosa and forms a biofilm on it, the internal fungal cells rapidly run out of oxygen and the tight biofilm structure also prevents the free diffusion of external oxygen to the bottom of the Candida biofilm. As a result, the hypoxic environment created by reduced oxygen tension leads to macrophage death (106). In our group, paeonol combined with fluconazole or amphotericin B reduced the fungal burden of OPC mice, and decreased the hypoxic microenvironment and inflammatory response (105). Zhou et al. (107) reported that paeonol effectively enhanced the sensitivity of ovarian cancer cells to radiation by significantly downregulating VEGF and hypoxia-inducible factor (HIF)-1α. Amphotericin B suppresses migration and invasion of esophageal carcinoma in the hypoxic microenvironment by downregulating HIF-1α activity (108). Our results show that both monotherapy and combination therapy reduce HIF-1α expression *in vivo* to mitigate hypoxia-induced injury (105). Accumulating evidence implicates that one of the major innate defence counts on IL-17 mediated signaling which is mainly produced by natural Th17 (nTh17) cells and γδT cells and adjusted elaborately by IL-23 and HIF-1α (4). Compared to infected mice, paeonol in combination with fluconazole/amphotericin B can also corrected the abnormal expression of HIF-1α, IL-17a and IL-23 mRNA, thereby treating OPC (105). HIF-1α is an important transcription factor that activates innate immunity and regulates IL-17 production. Paeonol decreases HIF-1α expression leading to a decrease in IL-17A. And paeonol combined with fluconazole/amphotericin B significantly downregulated HIF-1α and effectively reduced IL-17 expression. These findings provide insights into the clinical application of paeonol as a sensitizer for fluconazole or amphotericin B in oral fungal diseases.

#### 3.4.4 Toxicity of paeonol

There are no systematic scientific reports on the *in vivo* toxicity of paeonol. The maximally tolerated dose of paeonol was found to be 5000 mg/kg in acute toxicity study in female rats. Paeonol was found to be safe at a dose of 50, 100 and 200 mg/kg in repeated dose toxicity study in male and female rats (109). It indicates that a suitable dose is required for the pharmacological action of paeonol to be reasonably effective.

### 3.5 Magnolol

Magnolol (5,5’-diallyl-2,2’-dihydroxybiphenyl) (The chemical structure of magnolol in **Figure 2E**) is a polyphenolic binaphthalene compound and a structural isomer of honokiol. *Magnolia officinalis* Cortex (*M. officinalis*), as a primary component of traditional Chinese medicine formulae, was widely used for the treatment of infectious diseases associated with pathogenic microorganisms, such as constipation, diarrhea, asthma, phlegm, and malaria (110). Magnolol and honokiol are isolated from the stem bark of a TCM *Magnolia officinalis* and have a variety of beneficial activities, including anti-inflammatory, antimicrobial, antioxidant, antiangiogenic, anticancer, neuroprotective, cardiovascular protective (111).

Magnolol also has antibacterial activity against *C. albicans*. Studies have found that magnolol inhibits the adhesion, conversion from yeast to hyphae, biofilm formation and development of *C. albicans*. The molecular mechanism of these effects are also linked to the Ras1-cAMP-Efg1 pathway, and the expression of genes associated with this pathway was significantly down-regulated following magnolol treatment (112). The cAMP signaling pathway is critical in regulating *C. albicans* morphogenesis and environmental perception (113). MAPK signaling pathways, including the PKC, Cek1 and HOG pathways, are also important in regulating *C. albicans* hyphae formation, biofilm formation and adaptation to cell wall stress (114). The study found that magnolol significantly inhibited the expression of genes related to virulence factors in *C. albicans*, including ALS1, ALS3, EFG1, etc (115). In addition, PKC pathway-related genes and Cek1 pathway-associated genes were also significantly down-regulated, suggesting that the inhibition of virulence factors in *C. albicans* by magnolol may be related to the PKC and CEK1/MAPK signaling pathways (115). Magnolol combined treatment with FLU has a synergistic effect on *C. albicans*. Magnolol increases the intracellular content of azole antifungal drugs by competing with substrates of the ATP-binding cassette transporter protein Cdr1p, which can inhibit *C. albicans* azole efflux and reverse multidrug resistance in *C. albicans* infections (116).

#### 3.5.1 Oropharyngeal candidiasis

Campus et al. (117) proved that subjects chewing sugar-free gum containing 1.4 or 2.8 mg of magnolol for 5 min 3 times a day (with the total daily intake of magnolol being 7 mg/day) showed beneficial effects on oral health, including a reduction in salivary mutans streptococci, plaque acidogenicity, and bleeding upon probing, and no adverse reactions were observed in the subjects. In the present study, the concentration of magnolol is relatively safe for topical administration, allowing for its clinical employment as topical agents, including magnolol-added lozenges, mouthwash, toothpaste, etc. This is an important reference value in the treatment of OPC (115) (**Figure 3D**). Although more high quality clinical trials are still needed to confirm the efficacy and safety of magnolol in the future.

#### 3.5.2 Ulcerative colitis

Magnolol has a protective effect against colon-related diseases. Magnolol restrained the expression of TNF-α, IL-1β and IL-12 *via* the regulation of NF-κB and PPAR-γ pathways. Magnolol also enhanced the expression of ZO-1 and occludin in DSS-induced mice colonic tissues (118). Based on the antibacterial activity of magnolol against *C. albicans*, studies have shown that magnolol has a good improvement effect on cell infiltration and damage of DSS-induced UC model in mice, and can significantly change the level of colonic proinflammatory cytokines (119). Therefore, magnolol has significant reference value in *C. albicans* colonization aggravated DSS-induced colitis in mice (**Figure 3B**). Abnormal activation of the canonical Wnt/β-catenin pathway and up-regulation of the β-catenin/T-cell factor (TCF) response to transcriptional signaling play a critical role early in colorectal carcinogenesis. It was found that magnolol inhibited the nuclear translocation of β-catenin and significantly suppressed the binding of β-catenin/TCF complexes onto their specific DNA-binding sites in the nucleus, thereby inhibiting the growth of colon cancer cells (120).

In addition, magnolol has an important reference value in the process of innate immune response. Peroxisome proliferator-activated receptors (PPARs) are known to exert a cytoprotective effect against cellular inflammatory stress and oxidative injury. Magnolol significantly upregulated the expression of PPARγ and suppress the expression of NF-κB signaling, cyclooxygenase2 (COX-2), and inducible nitric oxide synthase (iNOS) and the production of ROS in bronchoalveolar lavage fluid, resulting in improvement of lung edema and polymorphonuclear neutrophil infiltration (121). Endotoxins released by *Porphyromonas gingivalis* (*P. gingivalis*) are involved in the development of inflammation-induced periodontitis. In *P. gingivalis* LPS-stimulated macrophages, magnolol treatment remarkably inhibited the inflammatory responses evidenced by suppression of pro-inflammatory cytokine, prostaglandin E2 (PGE2), nitrite (NIT) formation, and the expression of iNOS and COX-2, as well as NF-κB activation accompanied by a significant elevation of Nrf-2 nuclear translocation and HO-1 expression/activity. and thereby promoting its clinical use in periodontitis (122).

#### 3.5.3 Toxicity of magnolol

After the safety and toxicological studies of magnolol and honokiol, there was significant accumulation of magnolol in serum, urine, and kidneys only in mice treated for 3 months (123). These effects were accompanied by increase in clinical parameters relevant to kidney function, such as serum creatinine, urea nitrogen, and serum albumin. There were also significant alterations of the kidney ultrastructure morphology in the mice treated for 3 mo–a further indication of the kidney impairment induced by chronic treatment with *M. officinalis* methanol extract (123). To investigate whether the consumption of products containing magnolol can potentially result in dangerous clinical outcomes, increasing efforts have been made to assess their effects on human CYP and UGT enzymes. The inhibition of UGTs by magnolol may be a potential mechanism that enhances the toxicity of drugs or other active compounds contained in the herbal preparation (124). Therefore, the safe use of magnolol also requires a combination of other factors.

### 3.6 Dioscin

As a steroidal saponin, dioscin (diosgenyl 2,4-di-O-α-L-rhamnopyranosyl-β-D-glucopyranoside) (The chemical structure of dioscin in **Figure 2F**) could be isolated from various kinds of vegetables and herbs, most of which belong to the family of Dioscoreaceae. Dioscin is a bioactive compound that exhibits a range of ethnopharmacological and physiological properties including anti-inflammatory, antioxidative, lipid-lowering, antiobesity, hepatoprotective, and anti-tumor activities (43).

*C. albicans* biofilms, a microbial lifestyle with complex structures and high resistance to antifungal agents. Studies have found that dioscin has inhibitory effects on biofilm formation and development of *C. albicans*, morphological transformation, adhesion and other virulence factors such as extracellular phospholipase, thereby exerting its antifungal effect (125). Similarly, in order to visualize the effect of dioscin on the cell membrane, Cho et al. (126) synthesized rhodamine-labeled giant unilamellar vesicles (GUVs), mimicking the outer leaflet of the plasma membrane of *C. albicans*. It was found that dioscin exerts a considerable antifungal activity by disrupting the structure in membrane after invading into the fungal membrane, resulting in fungal cell death.

#### 3.6.1 Ulcerative colitis

Similarly, dioscin has a therapeutic effect on DSS-induced colitis. Dioscin reduced DSS-induced disease activity index (DAI) increase, colon length shortening and colon pathological damage. In addition, Dioscin inhibited NF-κB, MAPK signaling and NLRP3 inflammasome pathway in DSS-induced colitis, resulting in protective effects (127). NLRP3 inflammasome is an innate immune receptor that mediates the assembly of inflammasome complexes in the presence of microbial ligands, and is related to the pathogenesis of IBD (128). There is growing evidence suggesting that inhibiting the activation of NF-κB pathway and NLRP3 inflammasome can effectively treat UC (129). Macrophages are essential for intestinal homeostasis and the pathology of IBD, and can produce inflammatory mediators such as TNF-α, IL-1β, IL-6 and nitric oxide (130). Dioscin also modulates the polarization of intestinal M1/M2 macrophages. M1 (classically activated macrophages) are characterized by bactericidal activity and pro-inflammatory properties. On the contrary, M2 (alternatively activated macrophages) show an anti-inflammatory phenotype (34, 35). Dioscin treatment made intestinal macrophages of colitis mice more prone to differentiate into M2 type (127). Given its inhibitory effect on *C. albicans*, Dioscin is a promising candidate for the treatment of DSS-induced colitis exacerbated by *C. albicans* (**Figure 3B**). Occupational exposure to crystalline silica particles leads to silicosis, which is characterized by chronic inflammation and abnormal tissue repair. Alveolar macrophages (AMs) play a crucial role in the process of silicosis. Dioscin promoting autophagy leads to reduced crystalline silica-induced mitochondria-dependent apoptosis and cytokine production in AMs, which may provide concrete molecular mechanism for the therapy of silicosis (131). These studies have contributed to provide insights into the development of dioscin as a therapeutic drug for a wider range of diseases.

#### 3.6.2 Alcoholic liver disease

Like paeonol, dioscin can also enhance the antitumour activity of epirubicin. RPV-modified epirubicin and dioscin co-delivery liposomes enhanced tumor targeting and accumulation in tumor sites, and inhibited VM channel formation, tumor angiogenesis, migration and invasion (132). Does dioscin then have a hepatoprotective effect, attenuating to some extent the hepatotoxicity of epirubicin? Studies have found that dioscin shows an excellent protective effect against ethanol-induced liver injury through ameliorating ethanol-induced oxidative stress, mitochondrial function, inflammatory cytokine production, apoptosis, and liver steatosis (133). Dioscin exhibited potent therapeutic effects against alcoholic liver fibrosis (ALF) *via* altering the TLR4/MyD88/NF-κB signaling pathway (134). Furthermore, candidalysin activates the MAPK/c-Fos/MAP kinase phosphatase 1 (MKP1) signaling pathway, leading to the production of pro-inflammatory cytokines such as IL-1α, IL-1β, and IL-6 in epithelial cells (78). These pro-inflammatory cytokines may further recruit immune cells and hepatocyte injury and may contribute to direct candidalysin-induced hepatocyte death. Candidalysin can induce and aggravate ALD. Although it has not been reported in the literature that Chinese herbs can directly improve ALD symptoms by acting on candidalysin, it has a certain regulatory effect on ethanol-induced liver injury through the antifungal activity of Chinese herbs, which provides a new insight for further research on the mechanism of Chinese herbs as a candidate drug against *C. albicans* in treating ALD (**Figure 3C**).

#### 3.6.3 Toxicity of dioscin

Study showing dioscin did exert hepatoprotective effects on mice and rats (135). Nonetheless, the toxicity of dioscin must be considered, if administered in large dose. Pharmacological activity studies of dioscin showed oedema and megakaryocytes in hepatocytes of mice injected with high doses of dioscin *via* tail vein, indicating its potential hepatotoxicity (136).

### 3.7 Sodium houttuyfonate

*Houttuynin* (decanoyl acetaldehyde, CH_3_[CH_2_]_8_CHCH_2_CHO), a fishy-smelling herbaceous and edible perennial plant with creeping rootstocks and swollen nodes, is mainly found in moist and shady areas in east Asia countries such as China, Japan and Korea (137). *Houttuynin* is the main active ingredient of H. cordata, is prone to be oxidized or polymerized, resulting in the loss or decrease of its activities. *Sodium houttuyfonate* (SH, CH_3_[CH_2_]_8_COCH_2_CHOHSO_3_Na) (The chemical structure of sodium houttuyfonate in **Figure 2G**), which is an adduct of *houttuynin* and sodium bisulfite, is more stable than *houttuynin* and retains the major pharmacological activities of *houttuynin*, while widely used in clinical practice (138).

Traditional antimicrobial drugs are prone to drug resistance with long-term use, and sodium houttuyfonate can also synergize with other antimicrobial drugs in addition to its own antimicrobial activity. Our research found that Sodium houttuyfonate inhibited the planktonic growth of a panel of *C. albicans* strains, with MIC90 (the minimal concentration for 90% inhibition, obtained by the Clinical & Laboratory Standards Institute-guided micro-dilution method) ranging from 32 to 256 μg/ml (139). The biofilm of *C. albicans* could also be inhibited by Sodium houttuyfonate, which was more effective than the conventional antifungal drug fluconazole. Moreover, Sodium houttuyfonate can also synergize with fluconazole *in vitro*, lowering the dose while enhancing the efficacy of fluconazole (140). Sodium houttuyfonate can also synergize with ethylenediaminetetraacetic acid disodium salt (EDTA-Na_2_) in suppressing both the planktonic and the biofilm growth of *C. albicans in vitro*, and Sodium houttuyfonate, as well as the combination, can promote the survival of mice infected with *C. albicans*, without causing obvious toxic reactions, suggesting the low toxicity and the efficacy of this combination (141).

#### 3.7.1 Ulcerative colitis

Sodium houttuyfonate has the characteristics of pleiotropy, bidirectional regulation and low adverse effects. Zhang et al. (142) found that sodium houttuyfonate maintained the intestinal barrier and attenuated the production of intestinal proinflammatory cytokines (TNF-α、 IL-1β、 IL-6) and inflammation-related enzymes (iNOS、 COX-2) by regulating the NF-κB signaling pathway, thereby providing protection for the intestine. Our group has shown that Sodium houttuyfonate can attenuate *C. albicans*-associated colitis through β-glucan exposure (143). Mice treated with the *C. albicans*-infected DSS group, intestinal inflammatory changes were manifested as thickening of the intestinal wall, disappearance of crypts, destruction of glands, mucosal degeneration and necrosis, and increased inflammatory cell infiltration, which were greatly reduced after SH treatment. Exposure to β-glucan activates innate immune cells (e.g. macrophages) to clear *C. albicans* and promotes recognition of *C. albicans* by macrophages through coordination between Dectin-1 and TLR-2/4 (143). The clearance of *C. albicans* results in reduced irritation of sensitized macrophages, decreased pro-inflammatory products and increased anti-inflammatory levels. Ultimately, symptoms of colitis lessen. Furthermore, our group found that *C. albicans* interference caused intestinal microbiota dysbiosis accompanied by an increase of some harmful pathogens including Klebsiella and Bacteroides, through 16S rRNA gene sequencing. The results showed that in UC mice inflicted by *C. albicans*, the administration of sodium houttuyfonate could greatly improve the pathological signs, weaken the oxidative stress and inflammatory response, and enhance the intestinal mucosal integrity (144) (**Figure 3B**). These findings suggest that sodium houttuyfonate might be an effective compound for the treatment of UC complicated by *C. albicans* overgrowth through maintaining gut microbiota homeostasis, thereby improving intestinal function.

#### 3.7.2 Oropharyngeal candidiasis

Likewise, our team has proven that sodium houttuyfonate can treat OPC (**Figure 3D**). In mouse OPC models, fungal biofilms induce hypoxic microenvironment leading to damage to the stratum corneum and papilla of the tongue. Nadine et al. (145) reported that *C. albicans* can induce a strong HIF-1α signal in keratinocytes, dermal capillaries, neutrophils, dermal lymphocytes and macrophages, and subcorneal neutrophils. The results of our group showed that SH combined with FLU administration can significantly inhibit fungal growth and reduced HIF-1α levels as well as the hypoxic niche (146). It has been mentioned in the treatment of OPC with paeonol that HIF-1α is a key regulator of IL-17 in OPC. In this report, after SH combined with FLU treatment, HIF-1α was inhibited, IL-17A mRNA expression was significantly downregulated, and HIF-1α protein levels were inhibited by exogenous IL-17A (146). In oral cavity, the ephrin type-A receptor 2 (EphA2) may be the primary sensor of exposed β-glucan (147, 148). Marc et al. (148) indicated that EphA2 might be indispensable for a maximal IL-17 response in OPC. After ligation between the exposed β-glucan and EphA2, the immune cells (such as neutrophils) are then activated in response to *C. albicans* in OPC (146). Therefore, the involvement of EphA2 and dectin-1 in the recognition of exposed β-glucans may be one of the therapeutic mechanisms for SH/FLU in OPC. This will provide a prospect for the clinical application of paeonol and sodium houttuyfonate in oral fungal infections and lay a good foundation for possible research and development of antifungal synergists.

#### 3.7.3 Toxicity of sodium houttuyfonate

Adverse reactions to sodium houttuyfonate use are less commonly reported. Allergy is an abnormal reaction of the body to an allergen. Histamine is responsible for many of the acute symptoms of allergic diseases. The study found that the ability of sodium houttuyfonate to bind to tissue proteins and to activate histamine H1 receptor indicates a risk for drug adverse reactions of sodium houttuyfonate (149). Therefore, sodium houttuyfonate needs to be considered in multiple aspects in clinical application.

## 4 Conclusions and future directions

The interaction between *C. albicans* and the host is key to determining the tolerance and prognosis of *C. albicans*-induced disease. In the process of the immune response, a variety of immune cells and immune mediators are involved, all of which are regulated by each other, forming a complex and delicate system of resistance to *C. albicans* infection. Several studies have shown that herbs and natural active substances can be a new source of antifungal treatment and have some therapeutic effects on *C. albicans*. In this review, the therapeutic effects on herbal medicine on different parts of the host of C. albicans infection and innate immunoregulation are highlighted. Despite the serious challenges facing current anti-*C. albicans* treatments, further advances in these herbs and natural active substances will undoubtedly provide valuable insights into our future understanding of *C. albicans* infections.

## Author contributions

NL and T-MW conceived and designed the study. M-YB drafted the manuscript. M-YB, ML, Q-RB, YY, HS and C-ZW searched and reviewed the literatures, and made the figures and tables. All of the authors critically reviewed and revised the manuscript. All authors have read and agreed to the published version of the manuscript. All authors contributed to the article and approved the submitted version.
